# Assessment of economic, thermal and hydraulic performances a corrugated helical heat exchanger filled with non-Newtonian nanofluid

**DOI:** 10.1038/s41598-021-90953-6

**Published:** 2021-06-02

**Authors:** Muhammad Ibrahim, Ebrahem A. Algehyne, Tareq Saeed, Abdallah S. Berrouk, Yu-Ming Chu, Goshtasp Cheraghian

**Affiliations:** 1 School of Mathematics and Physics, University of Science and Technology Beijing, Beijing, 100083 China; 2 Department of Mathematics, Faculty of Science, University of Tabuk, P.O. Box 741, Tabuk, 71491 Saudi Arabia; 3 Nonlinear Analysis and Applied Mathematics (NAAM)-Research Group, Department of Mathematics, Faculty of Science, King Abdulaziz University, P.O. Box 80203, Jeddah, 21589 Saudi Arabia; 4 Mechanical Engineering Department, Khalifa University of Science and Technology, Sas Al Nakhl Campus, PO Box 2533, Abu Dhabi, United Arab Emirates; 5 Center for Catalysis and Separation, Khalifa University of Science and Technology, PO Box 127788, Abu Dhabi, United Arab Emirates; 6 Department of Mathematics, Huzhou University, Huzhou, 313000 People’s Republic of China; 7 Hunan Provincial Key Laboratory of Mathematical Modeling and Analysis in Engineering, Changsha University of Science and Technology, Changsha, 410114 People’s Republic of China; 8 Technische Universität Braunschweig, 38106 Braunschweig, Germany

**Keywords:** Mechanical engineering, Energy infrastructure

## Abstract

Improved heat transfer efficiency with considering economic analysis in heating systems is an interesting topic for researchers and scientists in recent years. This research investigates the heat transfer rate (HTR) and flow of non-Newtonian water-Carboxyl methyl cellulose (CMC) based Al_2_O_3_ nanofluid in a helical heat exchanger equipped with common and novel turbulators using two-phase model. The requirements for dimensions and cost reduction and also energy saving in thermal systems are the main goal of this study. According to gained results usage of corrugated channel in helical heat exchanger has a considerable influence on thermal and hydraulic performance evaluation criteria (THPEC) index of helical heat exchanger and can improve the THPEC index. Thus, Re = 5000 is obtained as an optimum value, in which the maximum THPEC value is achieved. As it is found in this paper, in case of using novel heat exchanger instead of the basic smooth system, the thermal properties (by considering Nusselt number) increases about 210%, the hydraulic performance (friction factor) reduces about 28%, performance evaluation criteria index increases about 57% and the material consumption (in case of similar THPEC) decreases about 31%. In another word, with considering economic analysis for the basic and novel system which has same efficiencies, the novel one has lower length and consequently 31% lower material.

## Introduction

Various experimental, analytical and numerical investigations are published due to intensifying the heat transferred amount in heat exchangers. The key aim of these researches is improvement in the heat transfer coefficient (HTC) with the minimum pressure drop. Recent study has been concentrated on the two effective techniques for HTR increment used in the heat exchangers. Vortex generators have been used recently as a typical example of HTR enhancement technique to increase the overall HTC from the fluid or nanofluid flow surface through an upsurge in turbulent motion because of the strong turbulence intensity increase. Nanofluids, which are new type of heat transfer fluids (HTRs), have been used recently also in the heat exchangers as an excellent HTR enhancement technique due to its thermal and physical properties^1,2^.

Due to the crucial applications of corrugated thermal systems in the apparatus such as heat exchangers, the topic is considered by many researchers^1–27^. Arie et al.^1^ performed an investigation on a manufactured innovative polymer-composite heat exchanger for applications in dry cooling field. Their work pays attention on the thermal characterization and also design of an innovative heat exchanger manufactured by a new process. Their study especially establishes the increasable manufacturing influence in understanding potentially transformative heat exchanger technologies which can also be very problematic for achieving conventional-fabrication methods. Bezaatpour and Rostamzadeh^2^ in a numerical study observed HTR improvement of a fin and tube compact heat exchanger which is under magnetite filed and is filled with ferro-nano-fluid flow. In their study, the influence of a uniform exterior magnetic field on HTR of a heat exchanger filled with Fe_3_O_4_/water nanofluid was numerically studied. Abeykoon et al.^3^ designed compact heat exchangers numerically. Numerical and analytical data presented only a 1.05% change in the cooling performance of the hot fluid characteristic. The axial pressure reductions displayed positive correlations with both the pumping power request and the overall HTC. Their achieved results validate that numerical simulation can be talented for optimization, design and manufacturing of thermal systems.

Saleh et al.^4^ carried out a numerical study on wire finned heat exchanger using aluminum fumarate for adsorption heat pumps. They investigated the performance of a wire finned and finned microchannel heat exchangers numerically and experimentally. Their numerical method was confirmed using empirical data and represented an excellent agreement between numerical and experimental results. Hagen et al.^5^ investigated a new method to analysis the Rankine cycle using the generic heat exchanger models. Their findings displayed that employing the innovative analysis resulted in lower net power than using the thermodynamic analyses and this behavior is because of working fluid depending pressure reduction penalty in thermal systems. Larwa and Kupiec^6^ considered a horizontal heat exchanger using an analytical method for the heat transfer process. They realized that variations in ground averaged yearly temperature concerns just the subsurface layers in the initial period of exchanging operation.

Naicker and Rees^7^ studied a large borehole heat exchanger array. The main goal in their study was to introduce a reference data set for analysis of large borehole thermal systems and verification of numerical methods. Warner et al.^8^ studied a novel shallow bore ground heat exchanger. Hu et al.^9^ studied numerically and experimentally investigated a PCM solar air heater and its preheating efficiency. This system was designed to improve indoor air quality and thermal comfort conditions. They employed solar energy as a permanent pre-heated air source and reported their results numerically and experimentally. A numerical investigation on the thermal performance enhancement of ground air heaters using sand-bentonite as backfilling material was done by Agrawal et al.^10^. Their study also exposed that the factor of thermal performance deterioration surges with the operation duration. Maximum thermal performance deterioration factor is obtained for ground-air heat exchanger with dry soil at airflow velocity of 5 m/s after 6 h of continuous operation. Minimum thermal performance deterioration factor is observed for ground-air heat exchanger system with wet sand-bentonite. Wen et al.^11^ observed that the amplitude upsurge and wavelength reduction result in heat transfer area and larger flow length of the corrugated channel, as well as higher HTR. The corrugated channel improves HTR compared to smooth channel. A numerical investigation on airside characteristics of sinusoidal corrugated heat exchangers subject to large-diameter tubes with round or oval cross-section tubes were invetigated by Chu et al.^12^. The CFD technique has also been adopted to examine complete local heat transfer presentation. Moreover, Yu et al.^13^ used longitudinal turbulators in a new compound parallel flow shell-and-tube heat exchanger to evaluate turbulent heat transfer. Experimental, analytical, mathematical and numerical investigations showed that the baseline geometry results in the best thermal performance. The increase of the LVG height and LVG attack angle may enhance the THPEC.

Employing horizontal flat-panel ground heat exchangers in ground-coupled heat-pumps was investigated by Habibi et al.^14^ numerically. Their achieved data present that even though growing buried depth leads to intensification in the thermal characteristic, the improvement rate reduces sharply when the ground heat exchanger is more than ten meter wide. Kerme and Fung^15^ studied in a numerical study heat transfer simulation, examination and presentation study of a single U-tube borehole heat exchanger. Their obtained results may significantly decrease the devoted time for investigation and to more rapid determination the impact of different parameters on vertical single U-tube borehole heat exchanger performance. Additionally, their achieved data can be used as a reference for cooling and heating system optimization and design integrated with system of ground coupled heat pump.

Keshavarz Moraveji et al.^28^ examined the heat transfer distribution of non-Newtonian nanofluids (NNNFs) in a horizontal-smooth tube under the steady state heat flux, numerically. The studied NNNF included an aqueous solution based Al_2_O_3_ with various nanoparticles sizes. Their achieved data indicates that the nanofluid HTC and predicted mean Nusselt numbers upsurge in the most non-Newtonian base fluid viscosity values. Akbari et al.^29^ studies numerically fluid flow and heat transfer distribution of a NNNF through a two-dimensional channel with rectangular cross-section. Their achieved data demonstrated that solid particles adding with various diameters to the base fluid upsurges the HTR in studied microchannel. Minea^30^ analyzed the turbulent flow and heat transfer distribution of water based Al_2_O_3_ nanofluid with various volume fractions of nanoparticles in a tube with micro-size. Their results showed that the enhancement of nanofluid HTC is remarkable and has been raised significantly by increasing the nanoparticles volume fraction. Zeinali Heris et al.^31^ studied during a numerical investigation fluid flow and heat transfer distribution of nanofluid flow in a circular pipe. They found that the solid particles may lead to an enhancement in the HTR. In recent years, interesting and diverse studies have been conducted in these fields^32–41^.

It should be noted that in most aforementioned investigation, the impact of different ribs and corrugations on hydraulic and thermal behavior of base fluid or nanofluids flowing in ducts has been investigated employing single phase model^42,43^. However, the literature review shows that the effect of novel turbulators (with minimum pressure drop error) on the hydraulic and thermal performance of two-phase NNNF has rarely been described.

Thus, the novelties of this article are as follows:

- Description of the thermal and hydraulic behavior of nanofluids flowing in a heat exchanger with novel turbulators using ANSYS FLUENT 18^44^ software.
- Evaluation of the impacts of using dissimilar types of turbulators on thermal–hydraulic characteristics.
- Investigating the impacts of different values of φ and various nanoparticle diameters on thermal and hydraulic behavior.
- In the simulation, two-phase modelling for nanofluid is used.

## Numerical model

### Physical model

**Figure 1:**
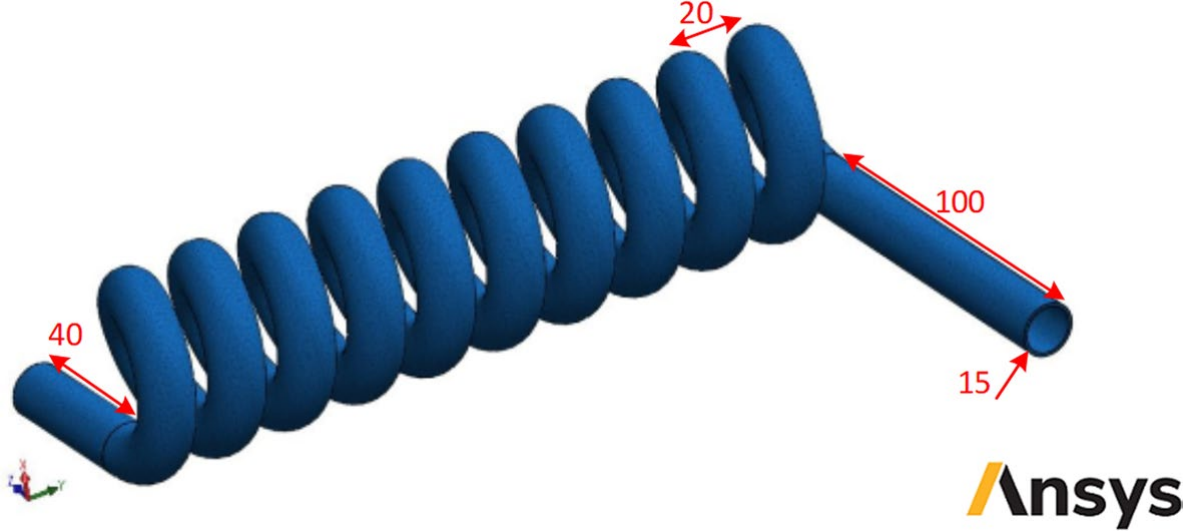
Schematic of studied smooth helical heat exchanger (Images used courtesy of ANSYS, Inc.).

**Figure 2:**
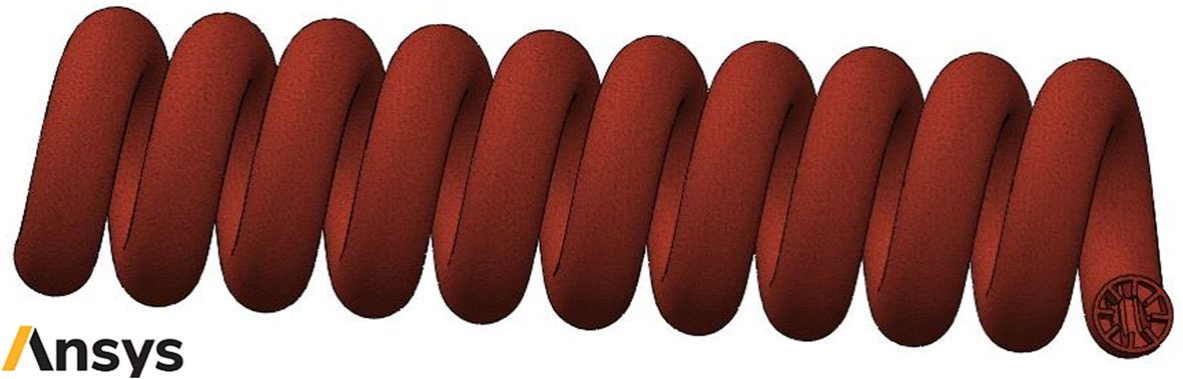
Schematic of studied helical heat exchanger with common turbulators (Images used courtesy of ANSYS, Inc.).

**Figure 3:**
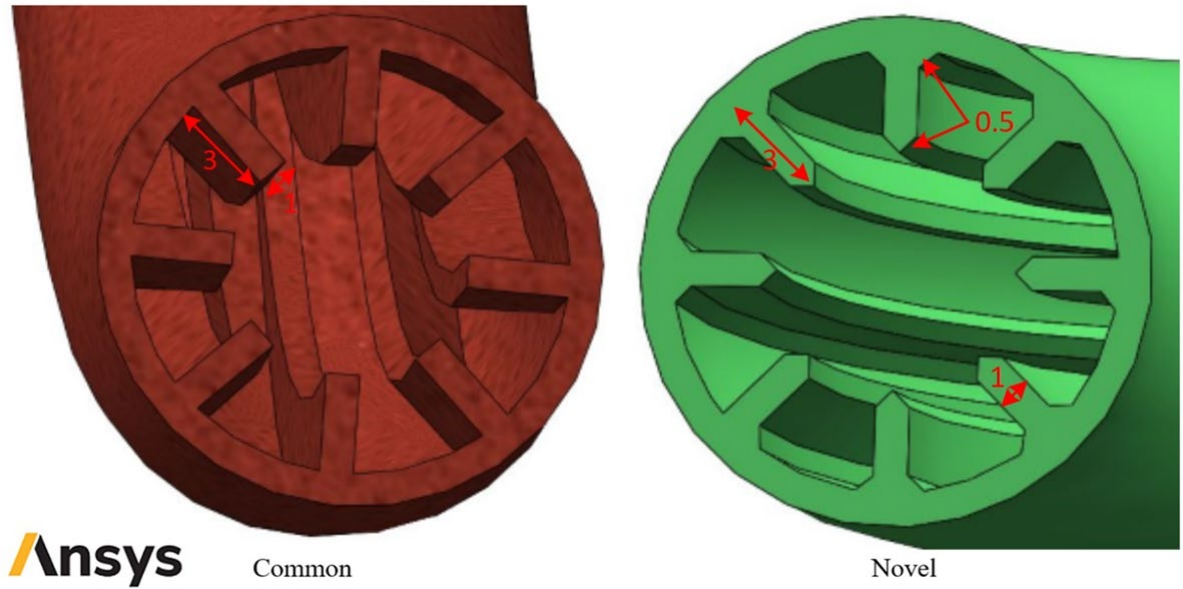
Schematic diagram of studied helical heat exchanger with different turbulators (Images used courtesy of ANSYS, Inc.).

The schematic of studied smooth helical heat exchanger has been presented in Fig. 1. Moreover, Figs. 2 and 3 show the schematic diagram of studied helical heat exchanger equipped with corrugations. In addition, the geometrical properties and configuration of turbulators are presented in this figure. The dimensional properties of the studied heat exchanger have been reported in Figs. 1, 2 and 3. The geometrical dimensions of the heat exchanger are the same for all considered configurations. In other words, the length and diameter of helical channel are similar for all studied models. Due to simplify the simulation procedure bellow assumptions have been considered^39^:

- The temperatures of walls are maintained at 400 K.
- There is no leakage between the different connections.
- The heat flux of the shell is ignored.
- The nanofluid properties are a function of temperature, volume fraction and particle sizes.
- Differences in hydric diameter are negligible.

### Conservation equations

The conservation equations system (energy, momentum, continuity) for two-phase NNNF in the helical heat exchanger may be inscribed as^39–41^:

1 $$\nabla .(\rho_{{{\text{nf}}}} V_{{\text{m}}} ) = \,0$$

${\rho }_{nf}$ is the nanofluid density and ${V}_{m}$ is the average velocity in each section.

2 $$\nabla .(\rho_{{{\text{nf}}}} V_{{\text{m}}} V_{{\text{m}}} ) = - \nabla P + \nabla .(\mu_{{{\text{nf}}}} \nabla V_{{\text{m}}} )$$

${\mu }_{nf}$ is the viscosity of nanofluid.

3 $$\nabla .(\rho_{{{\text{nf}}}} \,c\,V_{{\text{m}}} T) = \nabla .\,(k_{{{\text{nf}}}} \nabla T)$$

The supposition of dynamic viscosity behavior for the two-phase NNNF flow in solution field is observed by using of the non-Newtonian two-phase power-law method. The two-phase non-Newtonian power-law viscosity technique can be written as^42^:

4 $$\tau = K\dot{\gamma }^{{\text{n}}}$$

For viscosity and conductivity used equations in^45^**.**

The Reynolds stress is molded using standard *k-ε* turbulence model**.**

5 $$\left( { - \rho \overline{{u^{\prime}_{i} u^{\prime}_{j} }} } \right) = \mu_{t} \left( {\frac{{\partial u_{i} }}{{\partial x_{j} }} + \frac{{\partial u_{j} }}{{\partial x_{i} }}} \right)\,$$

6 $$\mu_{t} = \rho C_{\mu } \left( {\frac{{k^{2} }}{\varepsilon }} \right)\,$$

7 $$\frac{\partial }{{\partial x_{i} }}[\rho ku_{i} ] = \frac{\partial }{{\partial x_{j} }}\left[ {\left( {\mu + \frac{{\mu_{t} }}{{\sigma_{k} }}} \right)\frac{\partial k}{{\partial x_{j} }}} \right]\, + G_{k} - \rho \varepsilon$$

8 $$\frac{\partial }{{\partial x_{i} }}[\rho \varepsilon u_{i} ] = \frac{\partial }{{\partial x_{j} }}\left[ {\left( {\mu + \frac{{\mu_{t} }}{{\sigma_{\varepsilon } }}} \right)\frac{\partial \varepsilon }{{\partial x_{j} }}} \right]\, + C_{1\varepsilon } \frac{\varepsilon }{k}G_{k} + C_{2\varepsilon } \rho \frac{{\varepsilon^{2} }}{k}$$

*G*_*k*_ is the turbulent kinetic energy generation rate and *ρε* is the dissipation rate and defined by:

9 $$G_{k} = \left( { - \rho \overline{{u^{\prime}_{i} u^{\prime}_{j} }} } \right)\left( {\frac{{\partial u_{j} }}{{\partial x_{i} }}} \right)\,$$

The coefficients Pr_*t*_ = 0.9, *σ*_*ε*_ = 1.3, *σ*_*k*_ = 1.0, *C*_1*ε*_ = 1.44, *C*_2*ε*_ = 1.92 and *C*_*μ*_ = 0.09 are selected as experimental coefficients in turbulence transport equation^39^.

Due to analyze the two-phase NNNF flow characteristics and HTR of several turbulators shapes in the helical heat exchanger, definite descriptions have been assumed as^41–49^:

The Reynolds number (Re) has been expressed as:

10 $${\text{Re}} = \frac{{\rho_{{{\text{nf}}}} V_{{\text{m}}} D_{{\text{h}}} }}{{\mu_{{{\text{nf}}}} }}$$

where ${V}_{m}$ is fluid mean velocity.

The mean predicted Nusselt number (${\text{Nu}}_{{{\text{av}}}}$):

11 $${\text{Nu}}_{{{\text{av}}}} = \frac{{hD_{{\text{h}}} }}{{k_{{{\text{nf}}}} }}$$

where ${h}_{f}$ and ${k}_{f}$ are respectively the mean predicted HTC and thermal conductivity of non-Newtonian base fluid.

The pressure drop between the inlet and outlet is:

12 $$\Delta {\text{p}} = p_{{\text{avg,inlet}}} - p_{{\text{avg,outlet}}}$$

The average friction factor is^33^:

13 $$f = \frac{2}{{\left( {\frac{L}{{D_{h} }}} \right)}}\left( {\frac{\Delta p}{{\rho_{{{\text{nf}}}} V_{m}^{2} }}} \right)$$

The Thermal and hydraulic performance evaluation criteria (THPEC) is employed to calculate the performance of turbulators to improve HTR assuming the accretion of pumping power. Hence, Nu_av_ and mean friction factor are considered as follows^41–49^:

14 $${\text{THPEC}} = \left( {\frac{{{\text{Nu}}_{{{\text{av}}}} }}{{{\text{Nu}}_{{\text{av,s}}} }}} \right)\left( {\frac{{\text{f}}}{{{\text{f}}_{{\text{s}}} }}} \right)^{{\frac{ - 1}{3}}}$$

where Nu_av_ and Nu_av,s_ are the predicted mean Nusselt number for helical channel equipped with turbulators or filled with two-phase NNNF and the smooth channel or channel filled with non-Newtonian base fluid. Also, *f* and *f*_*s*_ are the friction factor for channel equipped with turbulators or filled with two-phase NNNF and the smooth channel or channel filled with non-Newtonian base fluid, respectively.

HTR in helical heat exchanger occupied with NNNF for two-phase flow is obtainable as follow^39^:

15 $$\dot{Q} = \dot{m}C_{ps} (T_{s,in} - T_{s,out} )$$

The commercial finite volume-based CFD code ANSYS FLUENT (version 18.1) has been employed in current investigation to perform the simulations. For the numerical approach, the velocity–pressure coupled equations are employed. Due to achieve a noteworthy accuracy, SIMPLEC algorithm is implemented. The maximum 10^−8^ error is considered for all parameters to economize the numerical procedure. The *Eulerian-Eulerian* single-fluid *Two-Phase Model* (TPM) are used to solve the governing equations. It should be noted that the phases linking is resilient, and particle prudently shadow the non-Newtonian two-phase nanofluid flow^46–56^.

The Eulerian–Eulerian two-phase method is based on the assumption that the base fluid particles and nanoparticles move at different speeds but have the same temperature. In fact, in this method, two momentum equations and one energy equation are solved.

For fluid boundary layer influences simulating near walls, solution the Navier–Stokes relations employing evaluate skin friction and the *k*–ε turbulence model in fluid fields a wall function approach has been employed in Launder and Spalding^57^ approach. However, the ANSYS FLUENTs Flow Simulation (SWFS), instead of using logarithmic profile, employs Van-Driest’s profiles. Besides, a *Two-Scale Wall Functions* (2SWF) technique has been implemented to express a turbulent and fluid boundary layers profile compared to Driest^58^ model. More than ten the laminar boundary layers simulation has been completed employing Navier–Stokes equations for cells quantities. Driest^58^ and Van Driest mixing length employs following dependency of the dimensionless longitudinal velocity *u*^+^ on the dimensionless wall distance *y*^+^ to precede turbulent boundary layers from the ANSYS FLUENTs Flow Simulations.

16 $$u^{ + } = \frac{u}{{\sqrt {\frac{{\tau_{w} }}{\rho }} }}\int\limits_{0}^{{y^{ + } }} {\frac{2d\eta }{{1 + \sqrt {1 + 4K^{2} \eta^{2} \left( {1 - e^{{ - \frac{\eta }{{A_{v} }}}} } \right)^{2} } }}}$$

where *A*_*v*_ is the Van Driest coefficient and is equal to 26. Moreover, *K* is the Karman constant and is equal to 0.4504. The diffusive heat flux and be determined by the following equation^51^:

17 $$q_{i} = \left( {\frac{\mu }{\Pr } + \frac{{\mu_{t} }}{{\sigma_{C} }}} \right)\frac{\partial h}{{\partial x_{i} }} \, i = 1,2,3$$

in which, *h* refers to the thermal enthalpy, ${\sigma }_{c}$ is the Stephan-Boltzmann constant and is equal to 0.9. Moreover, Pr indicates the Prandtl number. It must be mentioned that available equations express both regimes of laminar and turbulent flows. For purely laminar flow, *μ*_*t*_ and *k* are zero. The employed boundary conditions for two-phase non-Newtonian nanofluid HTR and flow distribution simulation are expressed as:

1. Total solid domains are known as non-slip conditions.
2. The shell wall is assumed adiabatic.
3. The velocity inlet boundary condition has been set for the inlet section of the channel.
4. Pressure outlet boundary condition is applied to the channel outlet.

**Figure 4:**
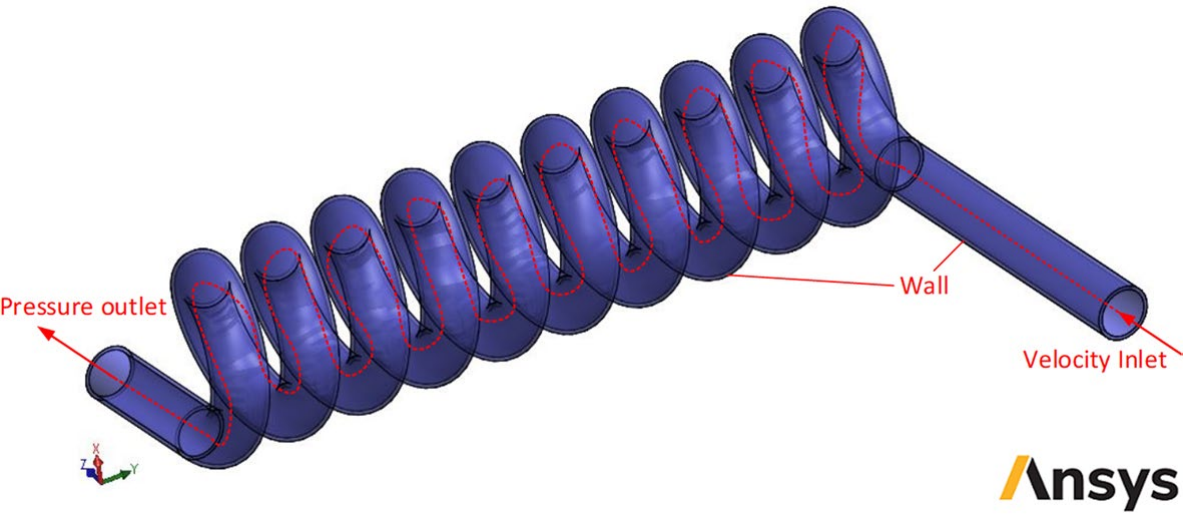
Schematic diagram of boundary conditions in studied helical heat exchanger (Images used courtesy of ANSYS, Inc.).

The inlet fluid temperature for the helical heat exchanger kept constant at 300 K, while the outlet pressure of nanofluid is kept at 100 kPa. The non-Newtonian HTR is H_2_O 99.5%:0.5% CMC. To achieve the most efficient NNNF in the current study, Al_2_O_3_ nanoparticles are added to the non-Newtonian base fluid in different volume concentrations of 1 to 4% with diameters of 20, 30, 40 and 50 nm (Table 1). Also, Fig. 4 shows schematic diagram of boundary conditions in studied helical heat exchanger.

**Table 1:** The thermophysical properties of the non-Newtonian base fluid and nanoparticles^59,60^.

Material	ρ (kg/m^3^)	c_p_ (kJ/kg·K)	k (W/m·K)	μ_0_ (Pa.s)
Pure Water	997.1	4.179	613 × 10^–3^	1 × 10^–3^
CMC	700	0.715	0.15 × 10^–3^	1.5
Al_2_O_3_	3970	0.765	40	$-$

### Model validation

**Figure 5:**
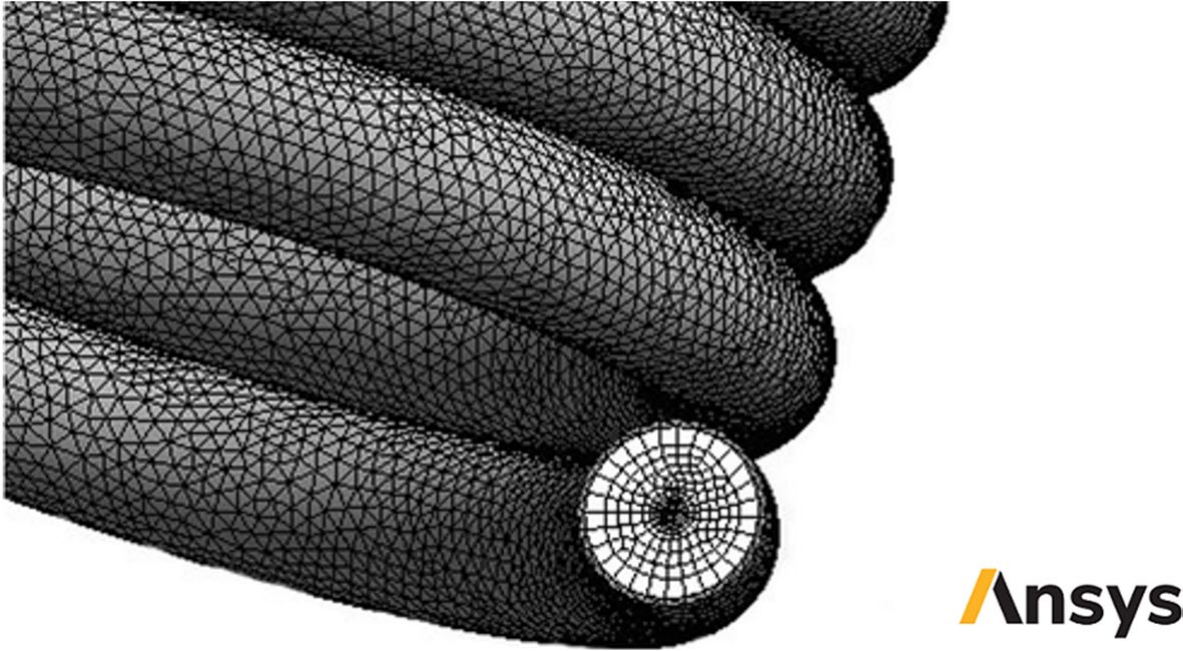
The images of unstructured tetrahedral grids in present study (Images used courtesy of ANSYS, Inc.).

The unstructured tetrahedral grid has been designated for the domain because of the complex space of two-phase flow NNNF near walls (see Fig. 5). Table 2 presents the results of the grid study for the conventional collector with water as working fluid using six grid resolutions. The Table demonstrates that the grid resolution of 3,132,341 can provide an excellent agreement between the numerical process cost and the accuracy.

**Table 2:** Grid independence test for sheet and tube collector.

No	Nodes	Nu
1	482,154	83.58
2	812,010	70.92
3	1,565,841	67.44
4	2,245,615	58.82
5	2,945,462	56.61
6	3,132,341	56.39
7	3,571,657	56.28

**Figure 6:**
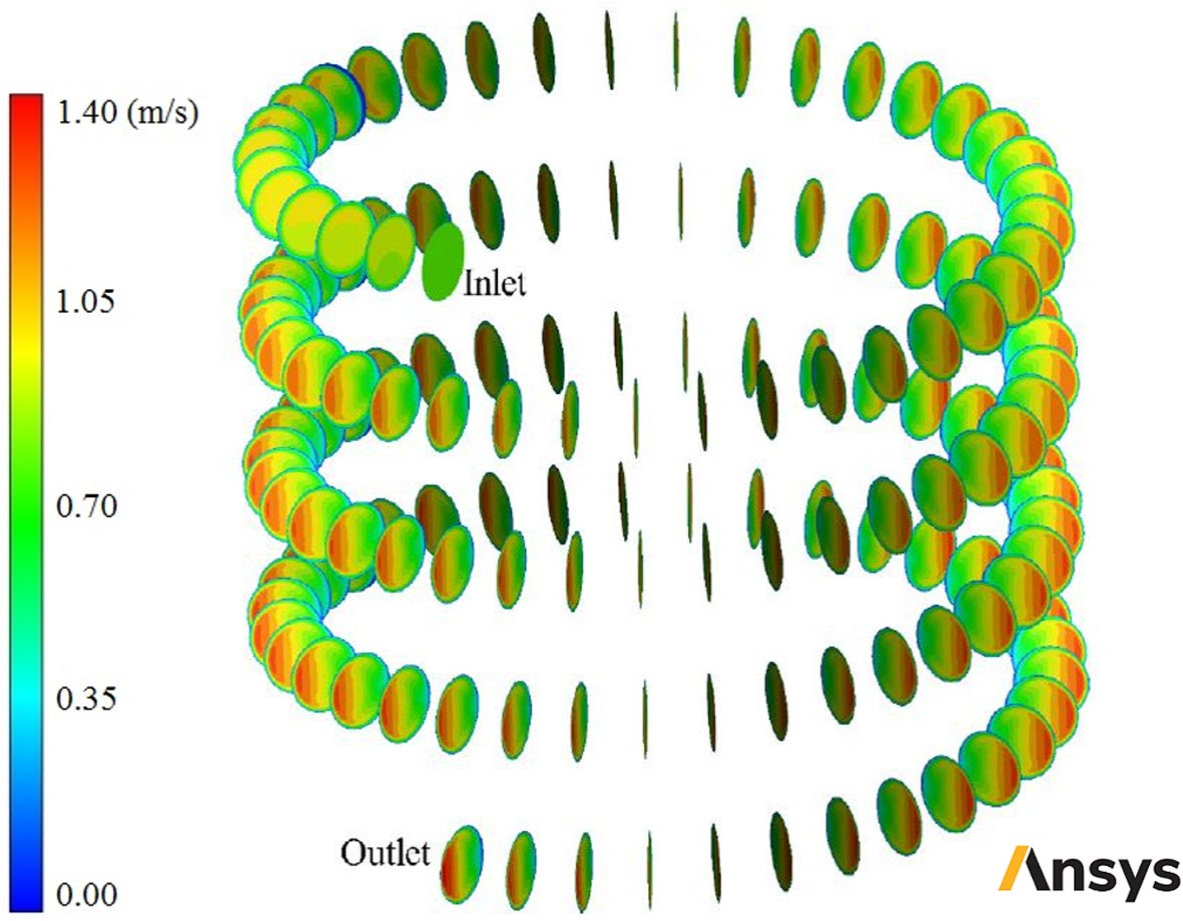
Velocity contours in some cross-sections of smooth-wall helical heat exchanger filled with two-phase NNNF (*φ* = 1% and *d*_*np*_ = 20 nm) (Images used courtesy of ANSYS, Inc.).

The CFD code verification was done by comparing previous numerical and experimental data and the current study. Figure 6 compares the current findings and the experimental results of Kim et al.^61^ and numerical ones of Karimi et al.^40^.

Table 3 also compares the present results with those of previous works^39^ and Dittus-Boelter equation (Nu = 0.023Re^0.8^ Pr^0.4^) for a tube saturated with pure water and nanofluid, indicating excellent agreement between the previous results and the present ones.

**Table 3:** Comparison between the present results and those of previous works^39^ and Dittus-Boelter equation.

	Dittus-Boelter	Mohammed et al.^39^	Present study
Re = 2400	21	18	19.6
Re = 3600	30	25.5	27.4
Re = 5600	42	40.5	39.7
Re = 9000	62	57.6	59

## Results and discussion

**Figure 7:**
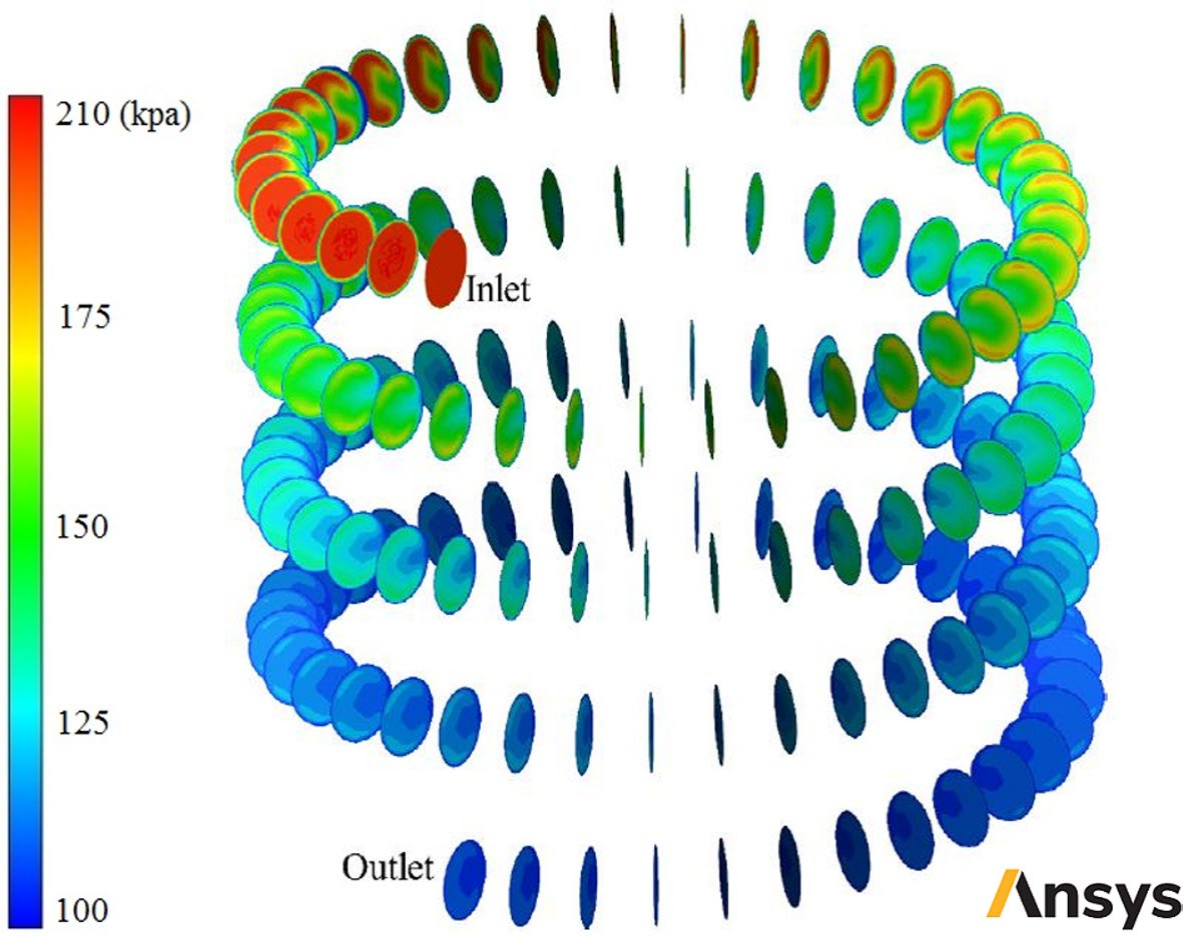
Pressure contours in some cross-sections of smooth-wall helical heat exchanger filled with two-phase NNNF (*φ* = 1% and *d*_*np*_ = 20 nm) (Images used courtesy of ANSYS, Inc.).

**Figure 8:**
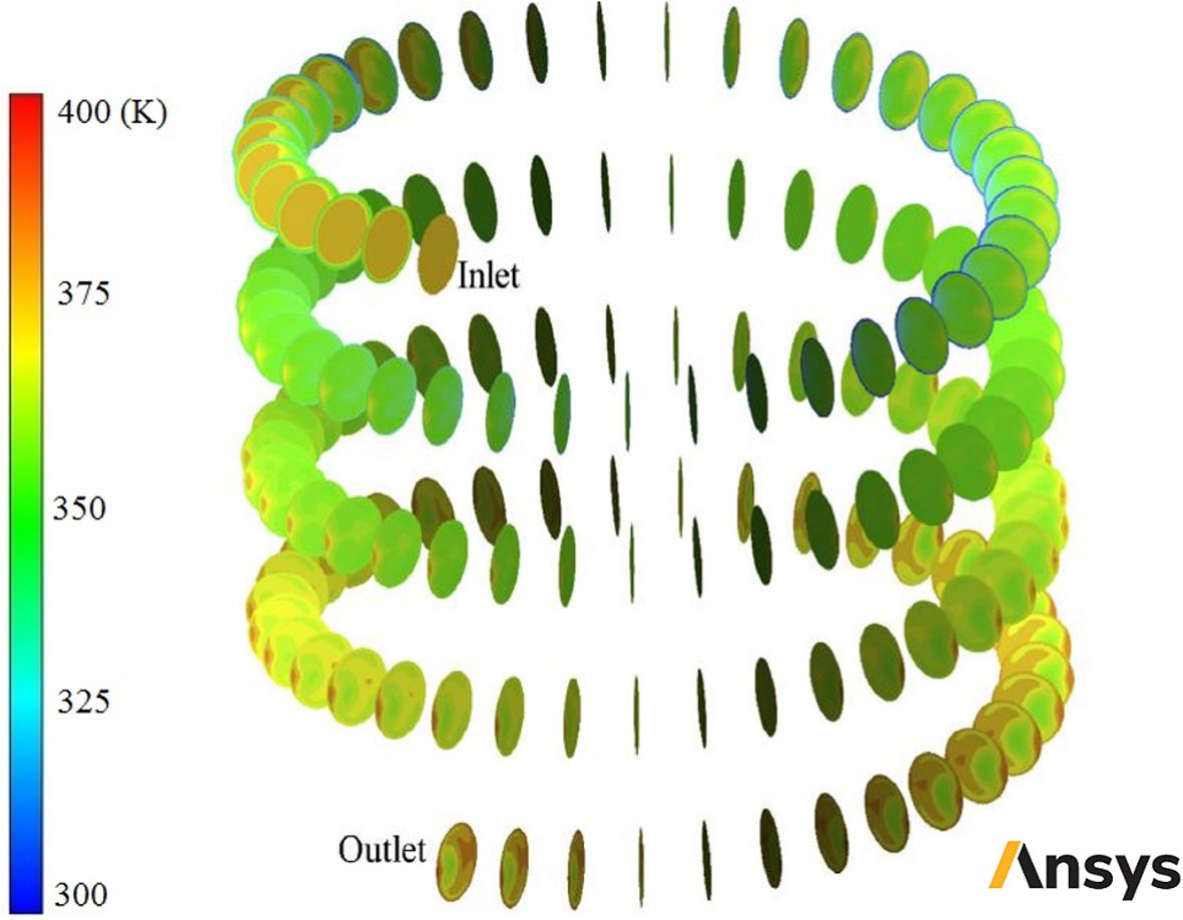
Temperature contours in some cross-sections of smooth-wall helical heat exchanger filled with two-phase NNNF (*φ* = 1% and *d*_*np*_ = 20 nm) (Images used courtesy of ANSYS, Inc.).

**Figure 9:**
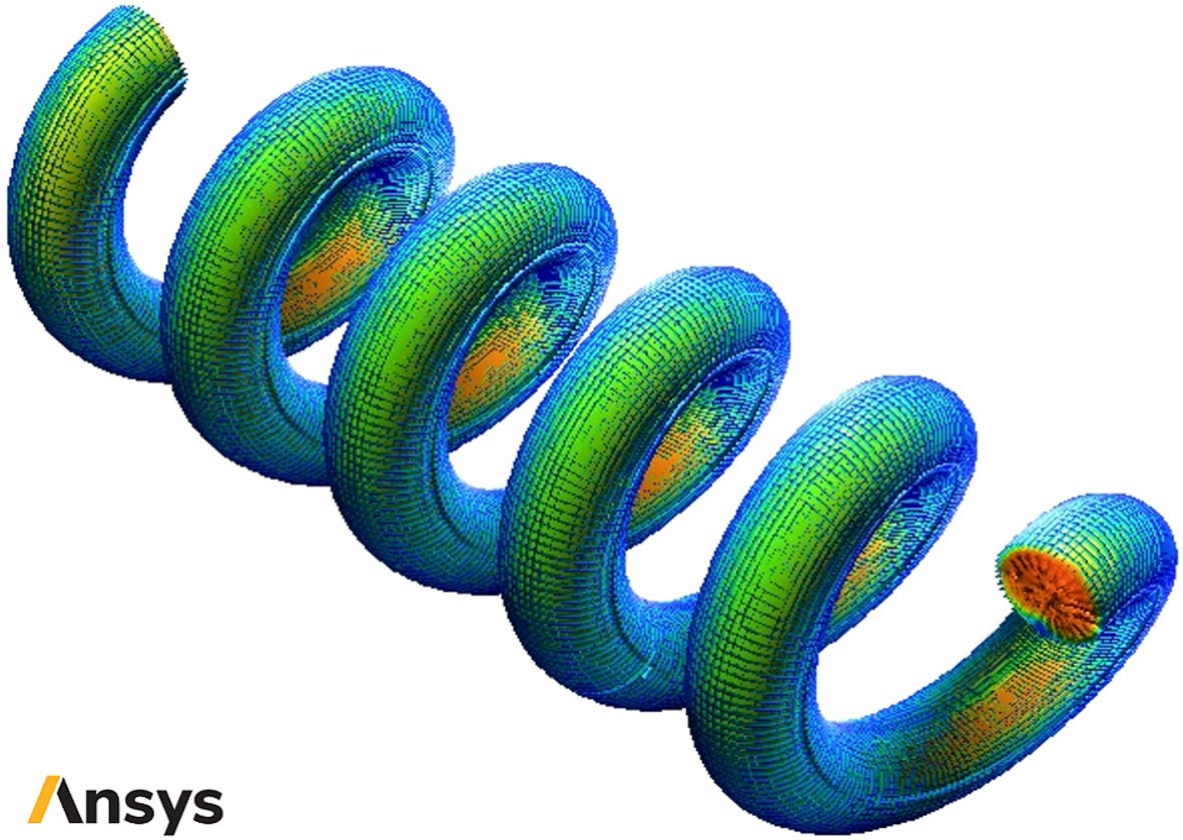
Velocity contour in smooth-wall helical heat exchanger filled with two-phase NNNF (*φ* = 1% and *d*_*np*_ = 20 nm) (Images used courtesy of ANSYS, Inc.).

In this section, usage of corrugated wall helical heat exchanger with different turbulators shapes is investigated and in then usage of nanofluid as heat transfer fluid with different φ and diameters is analyzed. In the first stage some contours are reported and the fluid flow and heat transfer field are analyzed. Figure 6 shows velocity contours in some cross-sections of smooth-wall helical heat exchanger filled with two-phase NNNF (*φ* = 1% and *d*_*np*_ = 20 nm). Figure 7 demonstrates pressure contours in some cross-sections of smooth-wall helical heat exchanger filled with two-phase NNNF (*φ* = 1% and *d*_*np*_ = 20 nm). Figure 8 illustrates temperature contours in some cross-sections of smooth-wall helical heat exchanger filled with two-phase NNNF (*φ* = 1% and *d*_*np*_ = 20 nm). Figure 9 represents velocity contour in smooth-wall helical heat exchanger filled with two-phase NNNF (*φ* = 1% and *d*_*np*_ = 20 nm).

### Optimum geometry

**Figure 10:**
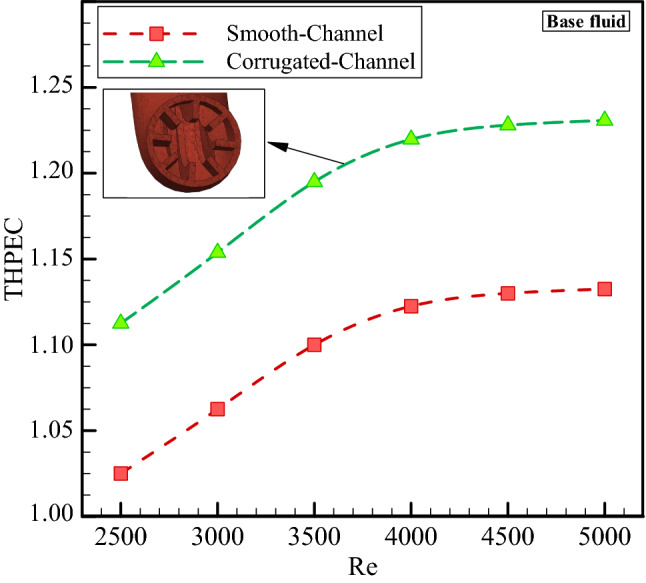
Geometrical effects on THPEC variation versus Re in helical heat exchanger filled with base fluid.

**Figure 11:**
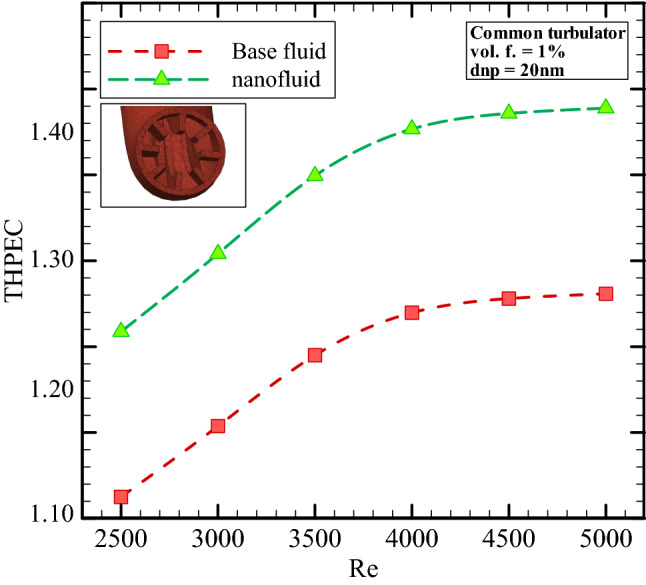
The THPEC index variation versus Re in helical heat exchanger filled with base fluid and nanofluid (*φ* = 1% and *d*_*np*_ = 20 nm).

Figure 10 represents geometrical effects on THPEC index variation versus Re in helical heat exchanger filled with base fluid. This figure shows that using corrugated channel in helical heat exchanger has a significant influence on THPEC index of heat exchanger and can improve the THPEC index in heat exchanger about 10%. Also, it is found that the THPEC index value always increases by increase of Re. Therefore, there is an optimum Re, in that the maximum THPEC value is achieved in is Re = 5000. It is clear that in case of using turbulators two parameters have increase: Nu_av_ and pressure reduction penalty. The vortexes and turbulent sub-layers increase the HTC in channel and also lead to more pressure drop in channel. But the THPEC index analyze the both above parameters with each other and when the THPEC is more than 1, usage of turbulators is in view point of thermal and hydraulic performance appropriate and efficient. The THPEC index can also help us to analyze the performance of using nanofluid in heat exchangers. If the THPEC will more than 1, employing nanofluid is in view point of thermal and hydraulic performance appropriate and efficient. Therefore, in the next step usage of nanofluid in heat exchanger is investigated. Figure 11 illustrates the THPEC variation versus Re in helical heat exchanger saturated with base fluid and nanofluid (*φ* = 1% and *d*_*np*_ = 20 nm) and equipped with common turbulators. This figure shows that using nanofluid (*φ* = 1% and *d*_*np*_ = 20 nm) in helical heat exchanger equipped with common turbulators has a considerable influence on the THPEC of heat exchanger and can improve the THPEC index in heat exchanger about 11%. Also, it is found that the PEC index value always increases by increase of Re. Therefore, there is an optimum Re, in that the maximum THPEC value is achieved in is Re = 5000. It is clear that in case of employing nanofluid two parameters have increase: Nu_av_ and pressure reduction penalty. The more conductive HTC of nanofluid increase the HTC in channel and the more dynamic viscosity of nanofluid lead to more pressure drop in channel. But the THPEC index analyze the both above parameters with each other and when the THPEC is more than 1, usage of nanofluid is in view point of thermal and hydraulic performance appropriate and efficient.

Figure 9 demonstrates the THPEC variation versus Re in three different helical heat exchangers filled with nanofluid (*φ* = 1% and *d*_*np*_ = 20 nm). This figure shows that using turbulators in helical heat exchanger has a considerable influence on the THPEC of heat exchanger.

It is clear that in case of employing turbulators two parameters have increase: heat transfer rate (Nu_av_) and pressure reduction penalty. The vortexes and turbulent sub-layers increase the HTC in channel and also lead to more pressure drop in channel. But the THPEC index analyze the both above parameters with each other and when the PEC is more than 1, usage of turbulators is in view point of thermal and hydraulic performance appropriate and efficient. Hence it should be noted that in case of using novel turbulators, the value of pressure drop penalty is less than in case of using common turbulators. Therefore, employing novel turbulators leads to more THPEC value than the other corrugated model. It is realized that in case of using novel configuration of turbulators, the THPEC values during all Re are more than other models, which is followed with common turbulators configuration and smooth channel. Also, it is found that the THPEC index value always increases by increase of Re. Therefore, there is an optimum Re, in that the maximum THPEC value is achieved in is Re = 5000.

### Optimum nanofluid

In this section different characteristics of nanofluid flow (nanoparticles volume fractions and diameters) are analyzed according to THPEC index.

Figure 10 shows the THPEC variation versus Re in smooth channel helical heat exchanger filled with nanofluid (*d*_*np*_ = 20 nm) in different φ. This figure shows that using nanofluid in higher volume fractions in heat exchanger with smooth channel leads to more THPEC of heat exchanger and can improve the THPEC index in heat exchanger about 8%.

In addition, it is found that the THPEC index value always increases by increase of Re. Thus, Re = 5000 is an optimum Reynolds number, in which the maximum PEC value is achieved. Employing nanofluid in higher volume fractions two parameters have increase: Nu_avg_ and pressure reduction penalty. The more conductive HTC of nanofluid in higher volume concentrations increase the HTC in channel and the more dynamic viscosity of nanofluid in higher volume fractions lead to higher pressure drop in channel. But the THPEC index analyze the both above parameters with each other and when the THPEC is more than 1, usage of nanofluid is in view point of thermal and hydraulic performance appropriate and efficient. For the nanofluid with diameter size of 20 nm and volume fraction of 4% the maximum values of THPEC are achieved for all investigated Re, which is followed with cases *φ* = 3%, 2% and 1%, respectively.

Figure 11 shows the THPEC variation versus Re in helical heat exchanger with common turbulators saturated with nanofluid (*d*_*np*_ = 20 nm) in different volume fractions. This figure shows that using nanofluid in higher volume fractions in heat exchanger equipped with common turbulators results in higher THPEC index of heat exchanger and can improve the THPEC index in heat exchanger about 12%. Also, it is found that the THPEC index value always increases with Re. Thus, Re = 5000 is an optimum Reynolds number, in which the maximum THPEC value is achieved. Employing nanofluid in higher volume fractions two parameters have increase: Nu_avg_ and pressure reduction penalty. More conductive HTC of nanofluid in higher volume concentrations increase the HTC in channel and the more dynamic viscosity of nanofluid in higher volume fractions lead to more pressure drop in channel. But the THPEC index analyze the both above parameters with each other and when the THPEC is more than 1, usage of nanofluid is in view point of thermal and hydraulic performance appropriate and efficient. For the nanoparticles with diameter size of 20 nm and volume fraction of 4% the maximum values of THPEC are achieved for all investigated Re, which is followed with cases *φ* = 3%, 2% and 1%, respectively.

**Figure 12:**
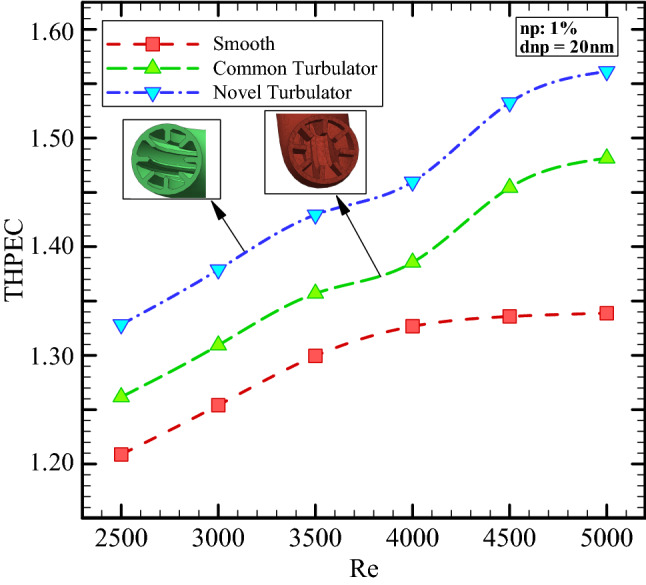
The THPEC index variation versus Re in three different helical heat exchangers filled with nanofluid (*φ* = 1% and *d*_*np*_ = 20 nm).

Figure 12 shows the THPEC variation versus Re in helical heat exchanger with novel turbulators saturated with nanofluid (*d*_*np*_ = 20 nm) in different volume fractions. This figure shows that using nanofluid in higher volume fractions in helical heat exchanger equipped with novel turbulators results in higher THPEC index of heat exchanger and can improve the THPEC index in heat exchanger about 13%. Also, it is found that the THPEC index value always increases by increase of Re. Thus, Re = 5000 is an optimum Reynolds number, in which the maximum THPEC value is achieved. Employing nanofluid in higher volume fractions two parameters have increase: Nu_avg_ and pressure reduction. The more conductive HTC of nanofluid in higher volume concentrations increase the HTC in channel and the more dynamic viscosity of nanofluid in higher volume fractions lead to higher pressure drop in channel. But the THPEC index analyze the both above parameters with each other and when the THPEC is more than 1, usage of nanofluid is in view point of thermal and hydraulic performance appropriate and efficient. For the nanofluid with diameter size of 20 nm and volume fraction of 4% the maximum values of THPEC are achieved during all investigated Re, which is followed with cases *φ* = 3%, 2% and 1%, respectively.

**Figure 13:**
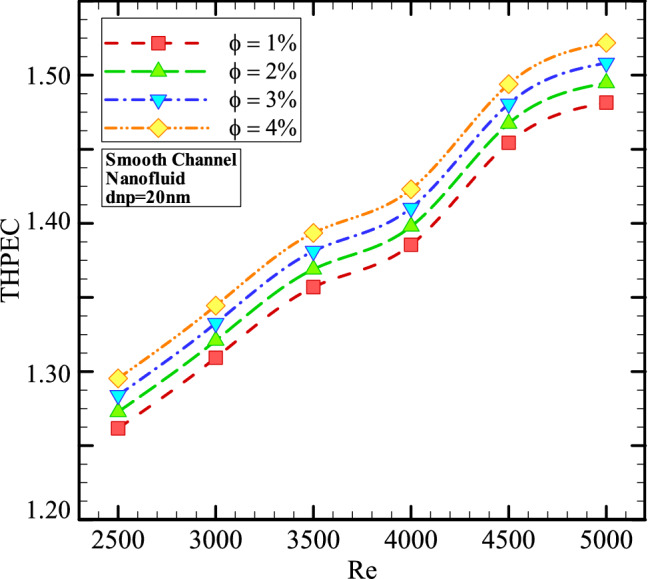
The THPEC variation versus Re in smooth channel helical heat exchanger saturated with nanofluid (*d*_*np*_ = 20 nm) in different volume fractions.

**Figure 14:**
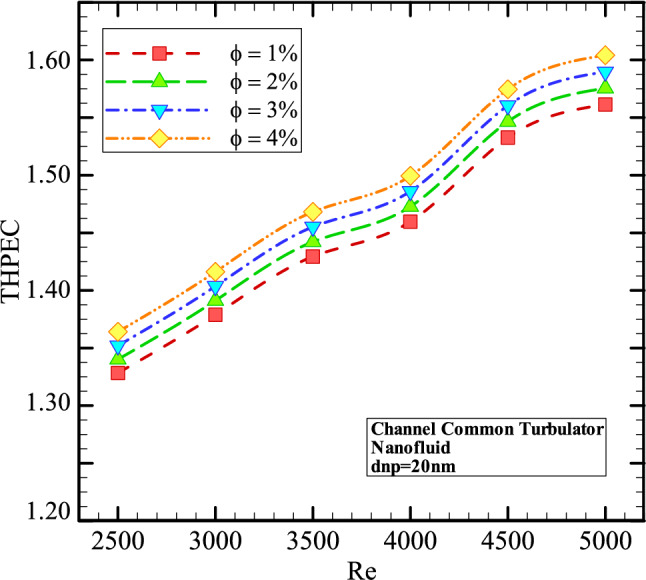
The THPEC variation versus Re in helical heat exchanger equipped with common turbulators saturated with nanofluid (*d*_*np*_ = 20 nm) in different volume fractions.

**Figure 15:**
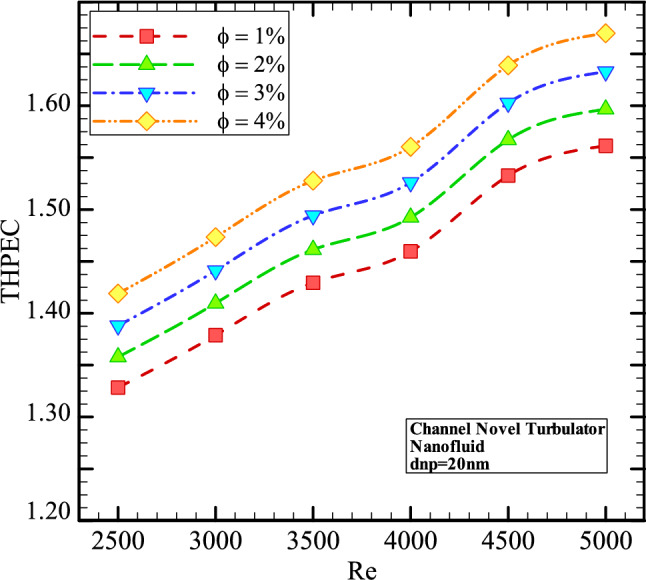
The THPEC variation versus Re in helical heat exchanger with novel turbulators saturated with nanofluid (*d*_*np*_ = 20 nm) in different volume fractions.

**Figure 16:**
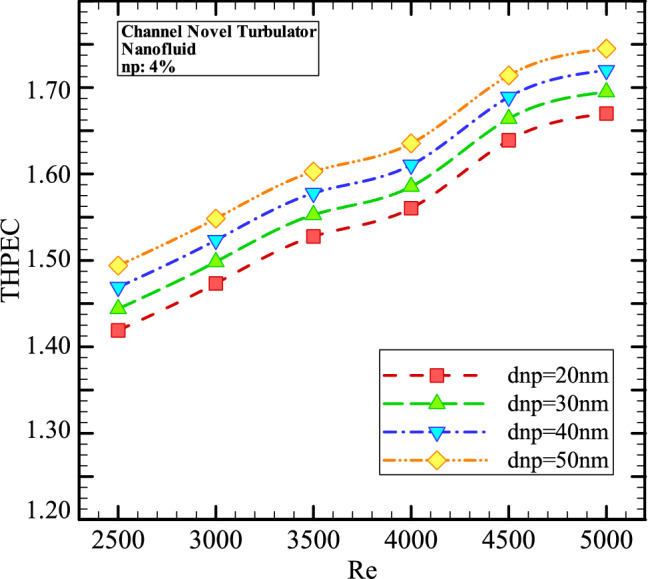
The THPEC variation versus Re in helical heat exchanger with novel turbulators saturated with nanofluid (*φ* = 4%) in different nanoparticles diameters.

Figures 13, 14, 15 and 16 show the THPEC variation versus Re in helical heat exchanger with novel turbulators saturated with nanofluid (*φ* = 4%) in different nanoparticles diameters. This figure reveals that using nanofluid in higher nanoparticles diameters in heat exchanger with novel turbulators leads to higher THPEC index of heat exchanger and can improve the THPEC index in heat exchanger about 15%. Also it is found that the THPEC index value always increases by increase of Re. Thus, Re = 5000 is an optimum Reynolds number, in which the maximum THPEC value is achieved. Employing nanofluid in higher nanoparticles diameters two parameters have increase: Nu_avg_ and pressure reduction penalty. The more conductive HTC of nanofluid in higher nanoparticles diameters increase the HTC in channel and the more dynamic viscosity of nanofluid in higher nanoparticles sizes lead to more pressure drop in channel. But the THPEC index analyze the both above parameters with each other and when the THPEC is more than 1, usage of nanofluid is in view point of thermal and hydraulic performance appropriate and efficient. For the nanofluid with diameter size of 50 nm and volume fraction of 4% the maximum values of THPEC are achieved for all investigated Re, which is followed with cases *d*_*np*_ = 40 nm, 30 nm and 20 nm, respectively^48^**.**

Finally, usage of H_2_O 99.5%:0.5% CMC/Al_2_O_3_ nanofluid in nanoparticle volume concentration of *φ* = 4% and nanoparticle diameter of *d*_*np*_ = 50 nm through helical heat exchanger equipped with corrugated channel with novel turbulators is suggested in paper as the optimum mode; with the maximum THPEC index at Re = 5000.

**Figure 17:**
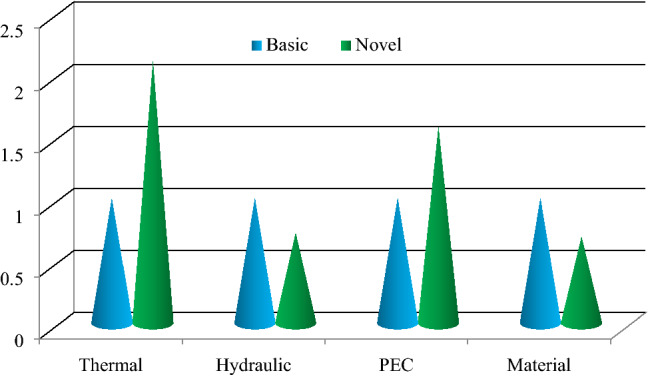
Economic and THPEC variation at Re = 5000 in helical heat exchanger with novel turbulators saturated with nanofluid (*φ* = 4% and *d*_*np*_ = 50 nm) in comparison with basic system.

Also Fig. 17 illustrates economic, the THPEC index variation at Re = 5000 in helical heat exchanger with novel turbulators saturated with nanofluid (*φ* = 4% and *d*_*np*_ = 50 nm) in comparison with basic system. This figure shows that using novel heat exchanger instead of the basic smooth system, the thermal properties (by considering Nusselt number) increases about 210%, the hydraulic performance (friction factor) reduces about 28%, performance evaluation criteria index increases about 57% and the material consumption (in case of similar thermal–hydraulic performance) decreases about 31%. In another word, for the basic and novel system which has same efficiencies, the novel one has lower length and consequently 31% lower material.

## Conclusion

Thermal–hydraulic analysis of a helical heat exchanger with novel turbulators saturated with two-phase NNNF has been done. The major obtained results are:

- Using corrugated channel in helical heat exchanger has a considerable influence on the THPEC index of heat exchanger and can improve the THPEC index in heat exchanger.
- Re = 5000 is an optimum Reynolds number, in which the maximum THPEC value is achieved.
- It is clear that in case of using turbulators two parameters have increase: heat transfer rate (Nu_avg_) and pressure reduction penalty.
- In case of employing nanofluid two parameters have increase: Nu_av_ and pressure reduction penalty.
- For the nanofluid with constant volume fraction, the maximum values of THPEC are achieved for all investigated Re for nanoparticles diameter of 50 nm, which is followed with cases *φ* = 3%, 2% and 1%, respectively.
- For the nanofluid with constant diameter size, the maximum values of THPEC are achieved during all investigated Re for volume fraction of 4%, which is followed with cases *d*_*np*_ = 40 nm, 30 nm and 20 nm, respectively.
- Usage of H_2_O 99.5%:0.5% CMC/Al_2_O_3_ nanofluid in nanoparticle volume concentration of *φ* = 4% and nanoparticle diameter of *d*_*np*_ = 50 nm through helical heat exchanger equipped with corrugated channel with novel turbulators is suggested in paper as the optimum mode; with the maximum THPEC index at Re = 5000.
- For the basic and novel system which has same efficiencies, the novel one has lower length and consequently 31% lower material.

## Symbols

*A*: Area of surface (m^2^)
*c*_*P*_: Specific heat (J/kgK)
*D*_*h*_: Hydraulic diameter (m)
*d*: Diameter of nanoparticles (nm)
*f*: Friction factor
*h*_*bf*_: Heat transfer coefficient of base fluid
$k$: Thermal conductivity (W/mK)
*k*_*np*_: Thermal conductivity of nanoparticle
*p*: Pressure (Pa)
*Q*: Heat flux (W)
Re: Reynolds number
Re_np_: Reynolds number of nanoparticle
*T*: Temperature (K)
*u*: Velocity
*V*_*m*_: Velocity

## Greek symbols

$$\alpha$$: Thermal diffusion
$\mu$: Dynamic viscosity (Ns/m^2^)
$$\rho$$: Density (kg/m^3^)
$\varphi$: Nanoparticles volume fraction

## Subscriptions

*bf*: Base fluid
*nf*: Nanofluid
*np*: Nanoparticle
